# Practical Chemistry and Pharmacy

**Published:** 1895-08

**Authors:** 

**Affiliations:** LaGrange, Ga.


### PRACTICAL CHEMISTRY AND PHARMACY.

EDITED BY HENRY R. SLACK, M.D., PH.M., LAGRANGE, GA.

### THE EFFECT OF FOOD ON THE ABSORPTION OF DRUGS.

Th. P. Malinin, in a study of the absorption of various medicaments in the empty and full stomach, came to the conclusion that absorption was very rapid when the stomach was empty, and that when food had been taken it was much slower. In one case it did not occur for eighty-five minutes, and in another for two hundred and fifteen minutes. Hydriode of potassium appeared in the saliva and urine, either at the same time, or three or five minutes earlier in the former, the excretion in both ceasing at the same period. This is probably due to the unequal action of the kidneys in different individuals. Sodium salicylate cannot be discovered in the urine by the ferric sesquioxide test. From the experiments of the author it appears that the excretion of medicinal substances corresponds to the rapidity of their absorption. The delay in absorption after a full meal is due to the mixing of substances with the food, and their separation from the stomach walls, rather than to the increased blood pressure in the organ incident to digestion.—*Jour. Med. Sciences.*

---

### COCAINE POISONING.

Schede reported at a recent meeting of the Hamburg Medical Society a case of cocaine poisoning by the injection of two grains of a ten per cent. solution of this drug into the urethra. Cyanosis, dilatation of the pupils and deep cornea supervened, followed by trismus and tetanus-like convulsions. Artificial respiration was begun, ether and camphor were subcutaneously injected, and nitrate of amyl was given by inhalation without effect on the symptoms. After twenty-five minutes Cheyne-Stokes respiration supervened and the cyanosis increased. Venesection produced no improvement. Under the inhalation of oxygen and the intravenous injection of 650 grains of salt solution, the patient finally rallied, the recovery being attended by transitory convulsions, and three hours elapsing before complete recovery of consciousness. In another case recently reported in the *Boston Medical and Surgical Journal*, death resulted from a urethral injection of six grains of a five per cent. solution of cocaine.—*Drug. Circular.*

---

### SYNTHESIS OF COFFEINE.

Professor Fisher of Berlin and his pupil, Lorenz Ach, have succeeded in forming coffeine by synthesis, their announcement appearing in the *A Kavenrische Berichte.* The steps of the synthesis occur as follows: Dimethyl-urea and malonic acid unite to form dimethyl-malonyl-urea. From this latter, by nitric acid, a nitrosi body is obtained, which, by reduction, produces dimethyluranyl, and this, combined with urea, yields dimethyl-pseudo-uric acid. From the latter dimethyl-uric acid is obtained, and from it theophyllin (dimethyl-xanthin), which, by methylization, gives us coffeine. The authors are hopeful of an early simplification of the process, and indeed indicate that the same is in sight.—*D. C. and Chem. Gaz.*

---

### TINCTURE OF CANTHARIDIN.

For the internal administration of cantharidin, Professor Liebreich recommends a tincture made as follows:

| | |
|---|---|
| Cantharidin | 0.1 gm. (1½ gr.) |
| Tinct. orange peel | 500 c. c. (16¾ fl. oz.) |

Dissolve the cantharidin by moderate heat in 300 c. c. (10 fl. oz.) of the tincture of orange peel; after cooling, add enough of the tincture to make the whole measure 500 c. c. (16¾ fl. oz.). The solution should be clear.

Of this tincture, or solution, 0.1 to 1 c.c. (1½ to 16 minims), according to circumstances, may be given in a wineglassful of water, some more water being taken after taking the medicine. This tincture of cantharidin, not to be confounded with tincture cantharidis, should be administered by the physician only, and not left in the hands of the patient.—*Drug. Circular.*

